# Supplementary material for: Prediction of interresidue contacts with DeepMetaPSICOV in CASP13
Source: Proteins. 2019 Jul 27;87(12):1092–9. doi: 10.1002/prot.25779 (PMC6899903; doi:10.1002/prot.25779)
Supplement: Supplementary file 1 — Table S1 Listing of all input features and their contributions to the DMP input feature tensor. For features defined on single residues, the feature values are striped horizontally and vertically to convert them into 2D feature maps with spatial dimensions of L × L, where L is the length of the target sequence. This causes such features to occupy twice the number of channels in the input tensor as compared to features defined on residue pairs. Table S2. List of dilation rates d for each of the 18 residual blocks in the DMP ResNet. A dilation rate of d = 1 produces regular, non‐dilated convolutions. Figure S1. Change in Top‐L/5 long‐range precision (in percentage points; y‐axis) on the CASP12 FM domains. The number of effective sequences (Meff eff) in the full‐length MSAs for each target are shown on the x‐axis. Positive y‐values indicate an improvement in precision when including the data augmentations. T0894‐D1 and T0888‐D1 (marked) showed significantly worse results when using the augmented model, though the full‐length MSAs for these targets were problematic. [file PROT-87-1092-s001.pdf]

Prediction of inter-residue contacts with DeepMetaPSICOV in CASP13

Shaun M. Kandathil, Joe G. Greener and David T. Jones

Supplementary information

Table S1. Listing of all input features and their contributions to the DMP input feature tensor. For features defined on single residues, the feature values are striped horizontally and vertically to convert them into 2D feature maps with spatial dimensions of $L \times L$, where L is the length of the target sequence. This causes such features to occupy twice the number of channels in the input tensor as compared to features defined on residue pairs.

Feature	Feature defined for single residues (1) or residue pairs (2)	Dimensionality per residue or residue pair	Channels occupied in input tensor
PSIBLAST Sequence profile	1	21	42
MI	2	1	1
MIp	2	1	1
Mean contact potential	2	1	1
PSICOV contact scores	2	1	1
FreeContact (mfDCA) contact scores	2	1	1
CCMpred (plmDCA) contact scores	2	1	1
PSIPRED secondary structure	1	3	6
Shannon entropy in MSA columns	1	1	2
SOLVPRED solvent accessibility	1	1	2
log(1 + sequence separation)	2	1	1
Sequence bounds (channel of ones)	2	1	1
DeepCov raw covariances	2	441	441
		**Total**	501

Table S2. List of dilation rates d for each of the 18 residual blocks in the DMP ResNet. A dilation rate of $d = 1$ produces regular, non-dilated convolutions.

Residual block	1	2	3	4	5	6	7	8	9	10	11	12	13	14	15	16	17	18
Dilation rate d	1	2	1	4	1	8	1	16	1	32	1	64	1	1	1	1	1	1

Impact of data augmentations

To determine the impact of the data augmentation procedures, we compared the precision values obtained when turning the data augmentations on or off. Figure S1 shows the change in Top-L/5 long-range precision obtained on the CASP12 free-modelling (FM) domains when using the data augmentation procedures. Contact predictions were made using the full-length multiple sequence alignments (MSAs) for each target, and the same MSAs used by MetaPSICOV2 in CASP12 were used for this analysis. For the model with all augmentations, we used predictions from a single model (instead of the ensemble of 5 models used in CASP13). Using the augmented model, precision values improve or show no change for 25 out of the 35 FM domains. For two domains, T0894-D1 and T0888-D1, the results are much worse when using the augmentations, although we noticed that the MSAs for these targets clearly had problems: for T0894, the portion of the MSA covering domain 1 had a high gap fraction throughout, and the MSA for T0888 only had 2 raw sequences (M_{eff} = 2). Given these observations, we opted to use the augmented model in CASP13 as it led to an overall improvement in contact precision on the majority of the CASP12 FM domains.

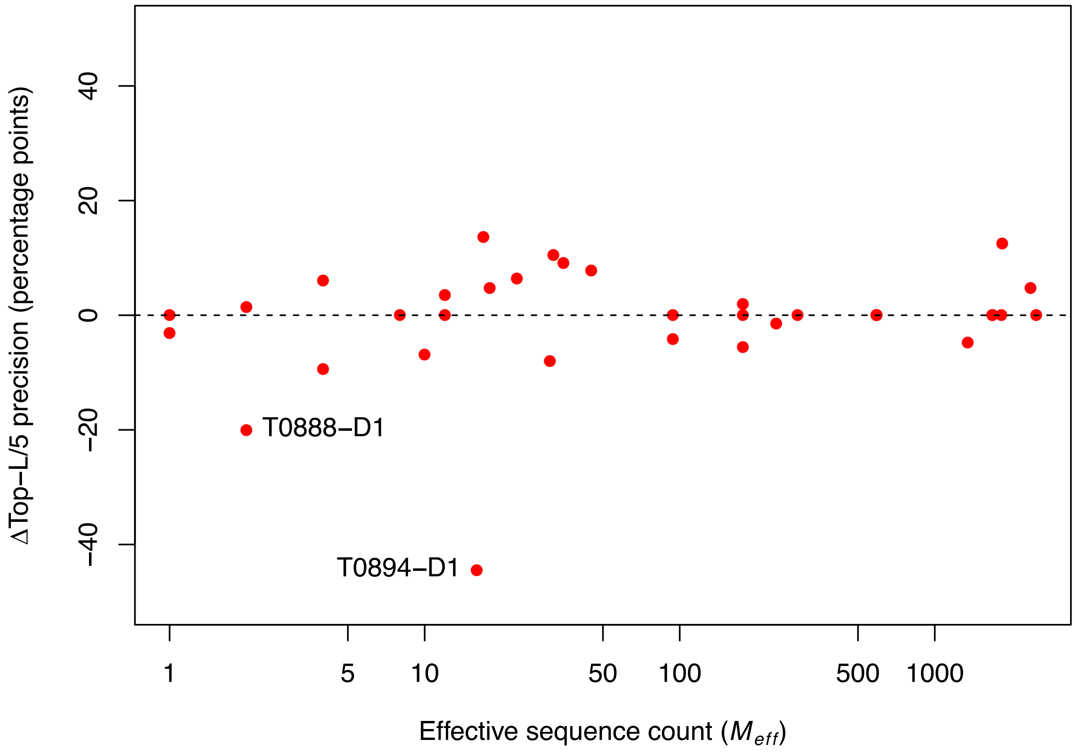

Figure S1. Change in Top-L/5 long-range precision (in percentage points; *y*-axis) on the CASP12 FM domains. The number of effective sequences (M_{eff}) in the full-length MSAs for each target are shown on the *x*-axis. Positive *y*-values indicate an improvement in precision when including the data augmentations. T0894-D1 and T0888-D1 (marked) showed significantly worse results when using the augmented model, though the full-length MSAs for these targets were problematic.
